# Case report: Bi-atrial thrombus after occlusion of atrial septal defect with acute cerebral infarction and pulmonary embolism

**DOI:** 10.3389/fcvm.2022.987538

**Published:** 2022-09-07

**Authors:** Wei Xiong, Li Tang, Wei Long, Jin Liu, Haibo Song

**Affiliations:** Department of Anesthesiology, West China Hospital, Sichuan University, Chengdu, China

**Keywords:** bi-atrial thrombus, atrial septal defect, transcatheter closure, acute cerebral infarction, pulmonary embolism

## Abstract

A 49-year-old man presented to the hospital with symptoms of acute cerebral infarction and pulmonary embolism who underwent transcatheter closure of atrial septal defect a year before. Transthoracic echocardiography showed a 13 × 9 mm hypoechoic mass attached to the left-atrial side of the device, which was suspected to be a neoplasm or thrombus. The patient was indicated for surgery after multidisciplinary discussion due to ineffective medical therapy and typical stroke and pulmonary embolism symptoms. Three-dimensional transesophageal echocardiography (3D-TEE) revealed left-atrial vegetation (21 × 16 mm) and right-atrial vegetation (8 × 6 mm) attached to the device, which was confirmed as thrombus by surgical separation and laboratory examination. This case highlights the importance of 3D-TEE and a multidisciplinary team in the diagnosis and therapy of device-related thrombus.

## Introduction

With improvements in echocardiography, cardiac catheterization, and device materials, transcatheter closure of atrial septal defect (ASD) has gradually replaced traditional thoracotomy surgery as the optimal treatment for patients with indications. However, long-term complications of catheter-based closure, especially device-related malposition, embolization, and thrombus formation, seriously threaten the life and health of patients (1). Bi-atrial thrombus formation-induced cerebral infarction and pulmonary embolism are rare but life-threatening late complications. Left atrial thrombus dislodges to the arteries of the brain, mesentery, and extremities, which can cause stroke, intestinal necrosis, and extremity ischemia; meanwhile, right atrial thrombus dislodges to the pulmonary artery and causes pulmonary embolism. Both conditions are common causes of acute cardiac arrest and sudden death.

## Case presentation

A 49-year-old man underwent transcatheter ASD closure with the Amplatzer^TM^ device (AGA Medical, Minnesota, USA) 1 year earlier and was continuously treated with aspirin (150 mg/day, orally; Huawei, Hebei, China) for long-term antiplatelet therapy. Six months before, the patient was hospitalized for dry cough and pleural effusion and was unfortunately misdiagnosed as pneumonia. One week after the previous admission, the patient was admitted to a high-level hospital for hemoptysis and right-limb weakness and was diagnosed with pulmonary embolism. After antithrombotic and symptomatic treatment, he recovered and was discharged home with long-term rivaroxaban (10 mg/day, orally; Qilu, Shandong, China) therapy.

At the latest admission, the patient presented with dizziness and pectoralgia, thus being diagnosed with acute cerebral infarction and pulmonary embolism. Physical examination revealed 3/5 strength in the right-limb without any other central nervous system abnormalities. ECG showed normal sinus rhythm. Blood tests showed a normal complete blood count and normal coagulation function.

**Figure 1:**
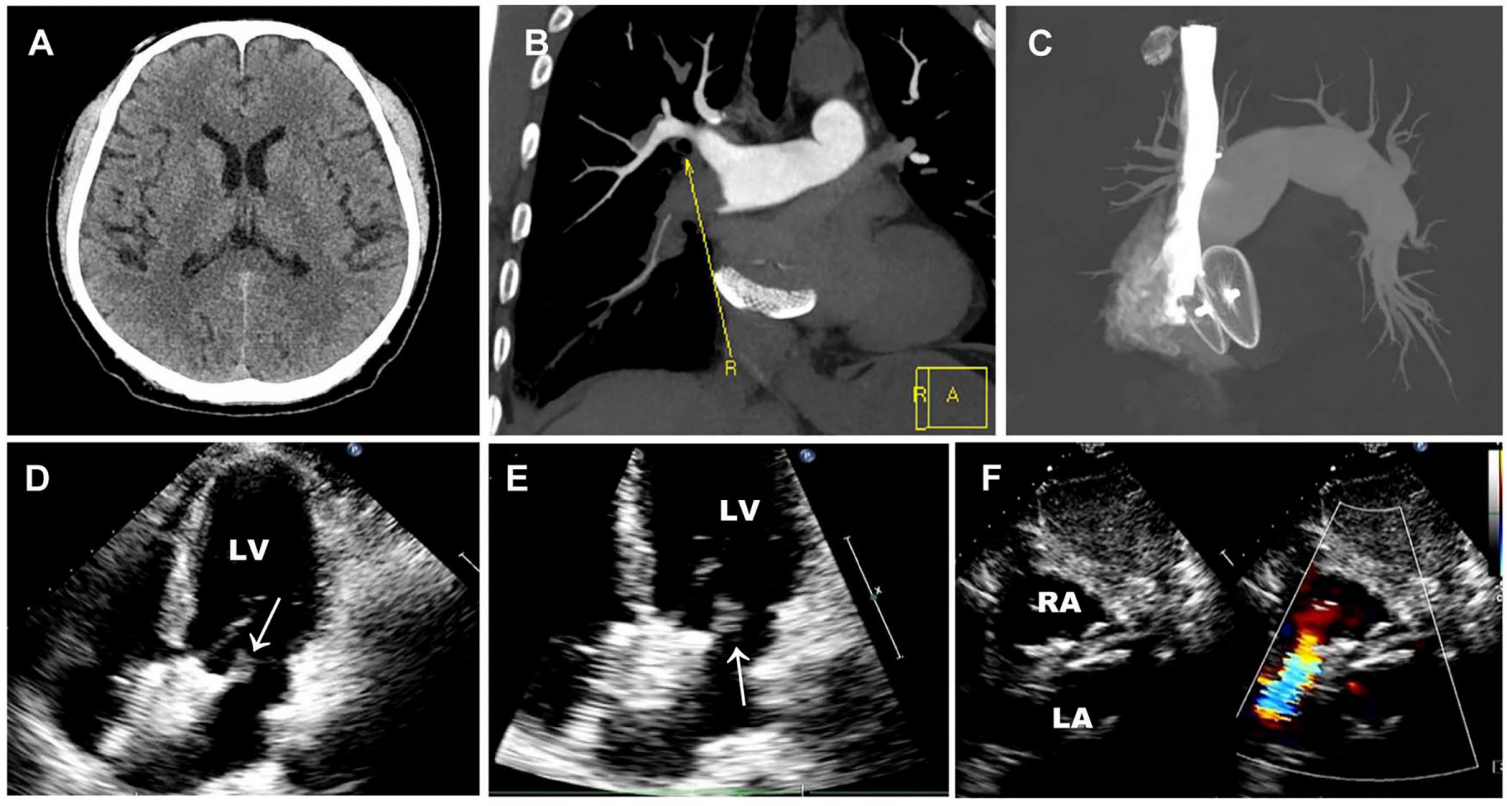
Preoperative imaging examination. **(A)** Brain CT manifested as post-infraction of cerebral lacunar with no other significant abnormalities. **(B,C)** Pulmonary CT angiography presented embolism in the distal of the right pulmonary artery and its branches. **(D–F)** Preoperative TTE showed a 13 × 9 mm thrombus floating in the left atrium without a shunt. White arrows represent thrombus.

**Figure 2:**
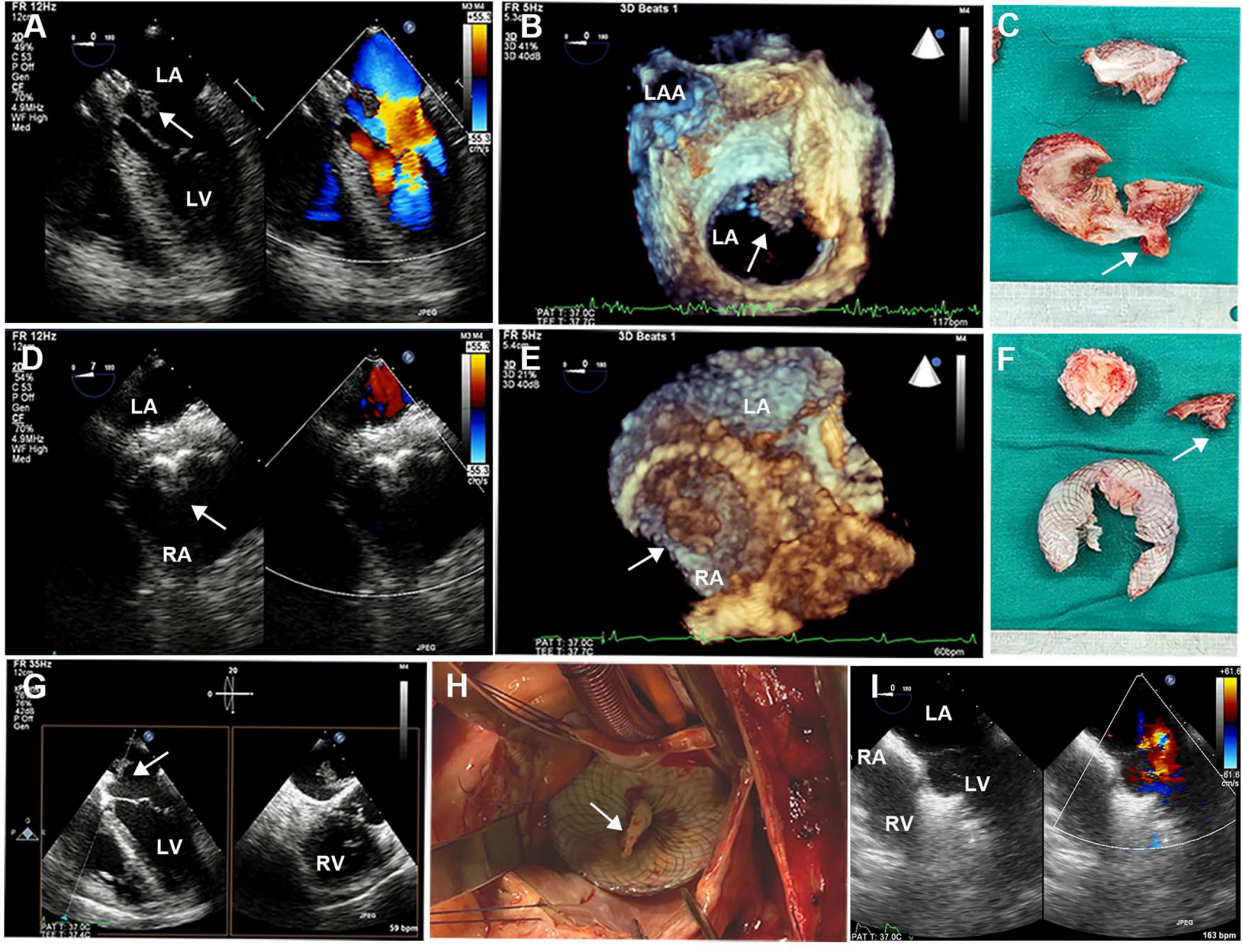
Intraoperative TEE and surgical images. **(A,D)** 2D-TEE color Doppler showed an ASD device with a double-sided thrombus. **(B,E)** 3D-TEE demonstrated a fan-like left-atrial thrombus attached to the device and a short rod-like right-atrial thrombus attached to the device. **(C,F)** Surgical resection of ASD device fragments with bilateral thrombus. **(G)** Biplanar TEE showed a giant thrombus floating in the left atrium. **(H)** Left atriotomy showed a white thrombus attached to the device. **(I)** Postoperative TEE showed continuous atrial septum without shunt and thrombus. TEE, transesophageal echocardiography; ASD, atrial septal defect. White arrows represent thrombus.

Brain CT manifested as post-infarction of cerebral lacunar with no significant abnormalities (Figure 1A). Pulmonary CT angiography presented an embolism in the distal of the right pulmonary artery and its branches (Figures 1B,C). Transthoracic echocardiography (TTE) showed a 13 × 9 mm hypoechoic mass attached to the left-atrial side of the device, which was suspected to be either a neoplasm or thrombus (Figures 1D–F). There was no shunt at the atrial level and left ventricular contraction was normal. Then transesophageal echocardiogram (TEE) was recommended to further examine this mass. Three-dimensional TEE revealed fan-like vegetation (21 × 16 mm) attached to the left-atrial side of the device and short rod-like vegetation (8 × 6 mm) attached to the right-atrial side (Figure 2; Supplementary Videos 1–3).

**Figure 3:**
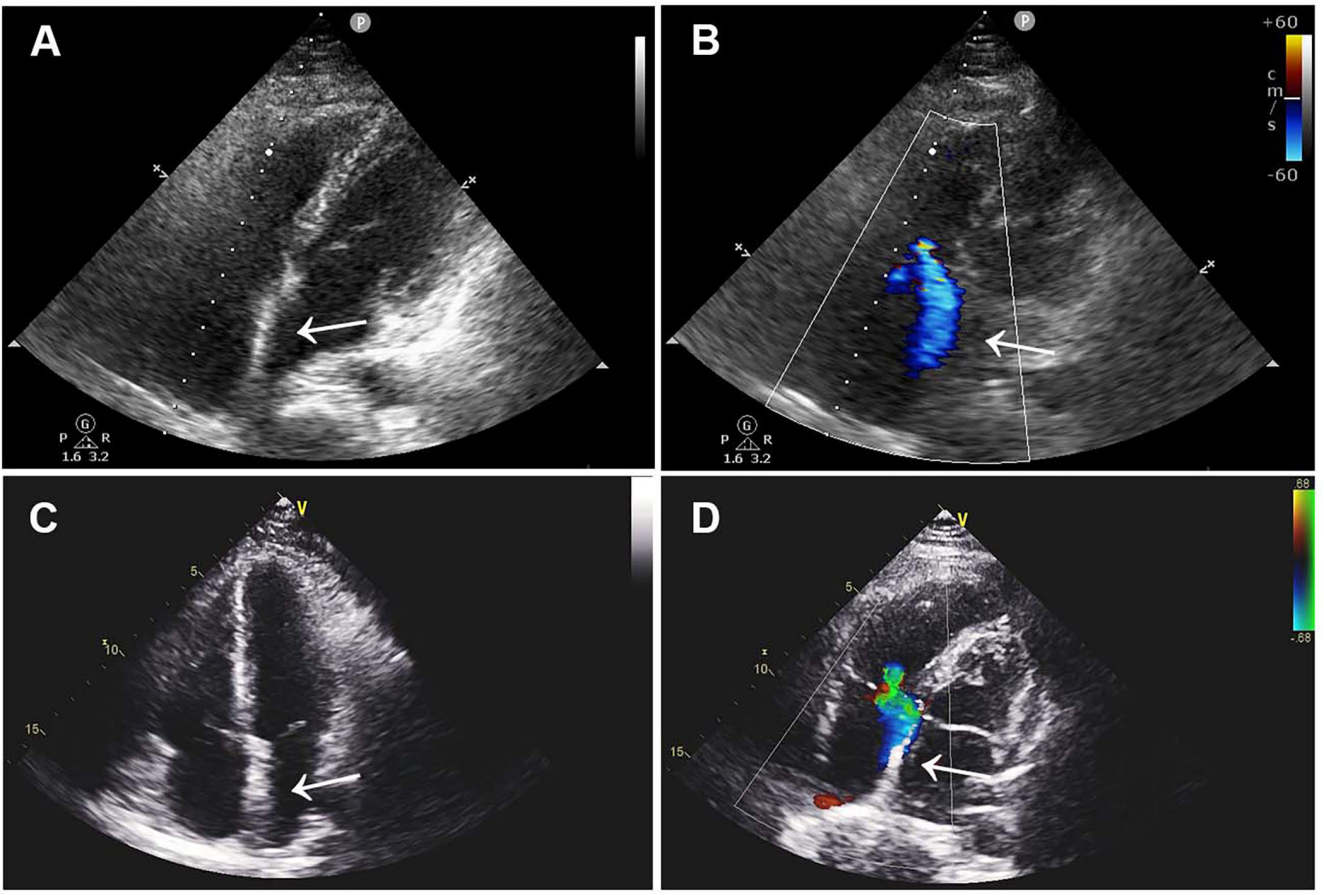
Postoperative TTE images. **(A,B)** TTE images 1 month after surgery showed continuous atrial septum without shunt and thrombus. **(C,D)** Three-month postoperative TTE images showed no novel shunt and no thrombus. TTE, transthoracic echocardiography.

The patient was treated with low-molecular-weight heparin (100 IU/kg/day, subcutaneously; Kelun, Sichuan, China) for anticoagulation and aspirin (200 mg/day, orally; Bayer, Beijing, China) for antiplatelet treatment under ECG and coagulation (APTT ratio) monitoring, followed by device removal and atrial defect repair under cardiopulmonary bypass. Following the operation, double-sided vegetations were found on the device, which was confirmed to be thrombosis by laboratory tests. TTE re-examination on the 5th day after the operation showed that the atrial septum was continuous and complete without shunts and thrombus. The patient fully recovered and was discharged home with long-term rivaroxaban (15 mg/day, orally; Qilu, Shandong, China) therapy. Three months after the operation, ultrasonography and laboratory examinations revealed that the atrial septum was structurally intact without shunts, and there was no thrombus found in the atrium, internal jugular vein, or deep veins of the lower extremities, along with normal blood counts and coagulation function (Figure 3).

## Discussion

Perioperative accidents and short-term complications of ASD occlusion are frequently reported, but mortality and morbidity rates are relatively low. The main reason is that hospitalized or newly discharged patients are highly alert to the disease and can receive timely and correct treatment under the guidance of professional doctors. Notably, device-related late complications (such as thrombosis and cardiac erosion) are rare but are often sudden and seriously life-threatening (2). Late thrombosis after device placement often causes ischemia of vital organs, including the lung, brain, intestine, and kidney, which is closely related to the patient's hypercoagulable state and inefficient anticoagulation therapy. The Amplatzer device had a significantly lower incidence of thrombi compared with other devices; the average time for detecting thrombi was 5 months after implantation, and most thrombi were located on the left side (3).

In this report, we present an unusual case of late bi-atrial thrombus after device-based closure of ASD with the Amplatzer device. Unfortunately, there are no definitive guidelines for cardiac thrombus management in patients with acute cerebral infarction and pulmonary embolism. A recent paper reported a case of intracardiac thrombus that crossed the patent foramen ovale (PFO) and caused an ischemic stroke and was treated with intravenous unfractionated heparin under close neurological monitoring (4). The patient in our case decided to undergo surgical resection and atrial septal repair by a multidisciplinary team, including cardiology, neurology, respirology, and hematology specialists. During the surgery, rivaroxaban antithrombotic therapy was switched to low-molecular heparin anticoagulation and aspirin antiplatelet treatment. Postoperative ultrasound found that there were no thrombi in the heart and limbs, except a few floating in the right internal jugular vein and subclavian artery.

Increasing evidence indicates that the device can be fully endothelialized 6 months after implantation, but under the influence of some predisposing factors such as infection, thrombosis may still form on the device during antiplatelet therapy. In this patient, early detection of clinical manifestations of cerebral infarction and pulmonary embolism should be excluded from atrial septal persistent shunts. Echocardiography revealed no atrial septal defect or intra-device shunt, and we speculated that the thrombus was attached to both sides of the device, causing pulmonary and systemic embolism, respectively. Pulmonary embolism occurred 6 months after aspirin antiplatelet therapy in this patient, which may be related to incomplete endothelialization of the device, causing right atrial thrombus formation and detachment into the right pulmonary artery. Following rivaroxaban antithrombotic therapy for 6 months, acute cerebral infarction still occurred, which may be due to the patient's lack of strict compliance during medication, resulting in multiple systemic thromboses and vital organ infarction. As noted above, these late complications are most likely due to the non-adherence to antiplatelet and antithrombotic therapy.

The Amplatzer device (St. Jude Medical, Minnesota, USA) and the Gore-Helex device (W.L. Gore, Newark, USA) are US Food and Drug Administration approval devices for percutaneous ASD closure, and the former is now the most frequently used in our practice. Krumsdorf and his colleagues (3) have previously reported the incidence of thrombus formation (20 of 1,000 cases) after catheter closure of intra-atrial shunts using six different devices, of which the CardioSEAL device (NMT Medical, Massachusetts, USA) had the highest rate of thrombosis (7.1% after 4 weeks), while the Amplatzer device (AGA Medical) had the lowest rate (0%). Late thrombosis associated with these devices is a rather unusual complication and is even rarer in the Amplatzer device. Giraldo-Gonzalez (5) reported a case of a 59-year-old man who presented with disorientation and right hemiparesis 8 years after implantation of the Amplatzer device, in which a left-sided thrombus (14 × 12 mm) attached to the device was identified. After 5 months of anticoagulation therapy treated with low-molecular-weight heparin, the thrombus and neurological symptoms disappeared in this patient.

Although TTE and CT have been widely used to detect intracardiac thrombus, 3D-TEE is considered to be more superior than both, thanks to its advantages, including real-time, clarity, and vividness (6). Compared with TTE, TEE can closely observe the cardiovascular system *via* the esophagus, which overcomes the interference of edema, emphysema, obesity, dressings, and mechanical ventilation. Meanwhile, TEE is more suitable for intraoperative guidance and timely evaluation as compared with CT. In addition, real-time 3D-TEE provides patients with a virtual reality heart model with high temporal and spatial resolution, allowing cardiac surgeons to identify structural changes and valvular lesions.

The European Society of Cardiology recommends antiplatelet therapy for 6 months and follow-up by a professional cardiology team for 2 years after device implantation (7), while the device manufacturer recommends clinical and echocardiographic follow-up at 1 week and at 1, 6, and 12 months after percutaneous closure. This case raises questions about the long-term safety of device-related complications and the relevance of prolonged clinical and echocardiographic assessments. It is noteworthy that clinicians should focus not only on standardized anticoagulation therapy and routine ultrasound follow-up but also on patient education and medication compliance.

## Data availability statement

The original contributions presented in the study are included in the article/Supplementary material, further inquiries can be directed to the corresponding author.

## Ethics statement

The studies involving human participants were reviewed and approved by Ethics Management Committee of West China Hospital of Sichuan University. The patients/participants provided their written informed consent to participate in this case study. Written informed consent was obtained from the individual(s) for the publication of any potentially identifiable images or data included in this article.

## Author contributions

WX, JL, and HS conceived the presented idea. WX, LT, and WL wrote the manuscript. WX made the videos. LT and WL drew the figures. JL and HS revised the manuscript. All authors have read and approved the final version of the manuscript.

## Conflict of interest

The authors declare that the research was conducted in the absence of any commercial or financial relationships that could be construed as a potential conflict of interest.

## Publisher's note

All claims expressed in this article are solely those of the authors and do not necessarily represent those of their affiliated organizations, or those of the publisher, the editors and the reviewers. Any product that may be evaluated in this article, or claim that may be made by its manufacturer, is not guaranteed or endorsed by the publisher.

## Supplementary material

Supplementary Video 1 Mid-esophageal four chambers biplanar view demonstrating a giant thrombus floating in the left atrium.

Supplementary Video 2 Three-dimensional TEE left atrial view showing a giant thrombus attach to the left-side of the device.

Supplementary Video 3 Three-dimensional TEE showing a right atrial thrombus attach to the device.
